# Revisional Laparoscopic Gastric Pouch Resizing for Inadequate Weight Loss After Roux-en-Y Gastric Bypass

**DOI:** 10.1007/s11695-015-1579-9

**Published:** 2015-01-21

**Authors:** Ibtisam Al-Bader, Mousa Khoursheed, Khalid Al Sharaf, D. Ali Mouzannar, Aqeel Ashraf, Abe Fingerhut

**Affiliations:** 1 Department of Surgery, Faculty of Medicine, Kuwait University, Safat, PO Box 24923, Jabriya, 13110 Kuwait; 2 Department of Surgery, Mubarak Al-Kabeer Hospital, Jabriya, Kuwait; 3 First Department of Surgery, Hippocration Hospital, University of Athens Medical School, Athens, Greece; 4 Section for Surgical Research, Clinical Division of General Surgery, Department of Surgery, Medical University of Graz, Graz, Austria

**Keywords:** Laparoscopy, Gastric pouch reduction, Gastric bypass, Revisional

## Abstract

**Background:**

Weight regain due to gastric pouch dilatation after Roux-en-Y gastric bypass (RYGB) is seen more frequently after long-term follow-up. We studied the feasibility and safety of laparoscopic pouch resizing (LPR) for dilated gastric pouch after RYGB associated with inadequate weight loss.

**Methods:**

From 1st June 2011 to 1st September 2013, patients who underwent LPR after failed RYGB were retrospectively compared and analyzed. Data included patient demographics, comorbidity, indication for revision, preoperative weight and BMI, operative time, hospital stay, conversion rate, mean follow-up, body mass index (BMI) loss, percentage excess weight loss (%EWL), reoperation rate, morbidity, and mortality.

**Results:**

Out of 170 revisional bariatric procedures, 32 LPR (27/5, F/M) were performed for dilated gastric pouch after RYGB. The mean age, preoperative weight, and BMI were 38.3 ± 9.3 years, 101.7 ± 22.8 kg, 38.8 ± 6.4 kg/m^2^, respectively. The median operative time and hospital stay were 100 min and 2 days, respectively. All pouch resizing procedures were carried out laparoscopically, with none requiring conversion to open surgery. The overall complication and reoperation rates were 15.6 and 3.1 %, respectively. There were no deaths. The mean follow-up was 14.1 ± 6.2 months. The mean postoperative BMI was 32.8 ± 7.3 kg/m^2^, and the median %EWL was 29.1 %.

**Conclusions:**

LPR is safe and can lead to adequate weight loss. However, long-term follow-up is needed to determine the efficiency and durability of this procedure.

## Introduction

Bariatric surgery has been shown to be most effective in achieving weight loss and improving comorbidities in morbidly obese patients [1–3], success being defined when ≥50 % excess weight loss (EWL) has been achieved, eventually associated with resolution of obesity-related comorbidities [4–6]. When patients were followed for at least 10 years, long-term failure rates, however, can be as high as 20.4 % after RYGB in morbidity obese patients and 34.9 % in super obese patients [7].

Inadequate weight loss is the primary indication for revisional bariatric surgery [8–10] procedures that include lengthening of the Roux limb, correction of large gastric pouch and stoma, and takedown of gastric-gastric fistula, the principal reasons for insufficient weight loss or weight regain [11].

Elongating the Roux limb has been shown to correct failed weight loss in super obese patients but requires nutritional support to prevent protein-calorie malnutrition, iron and vitamin deficiency [12, 13]. This has led to consider other options such as placing a band on the gastric pouch or conversion to biliopancreatic diversion and duodenal switch (BPD-DS) [14], shown to be more effective than Roux-en-Y bypass [15].

Revisional bariatric surgery, however, has been associated with high morbidity and mortality [16–18]. Less invasive procedures, such as the endoscopic transoral gastric plication (EGP) with the StomaphyX™ device (EndoGastric Solutions, Redmond City, WA), can reduce the size of the gastric pouch and the gastrojejunostomy. Although weight loss is maximal during the first 6 months with 19.5 % EWL at 1-year follow-up [19], weight loss has been reported to decrease on long-term follow-up: Both the pouch and stoma tend to regain their preprocedure size [20].

The literature on the benefits of LPR diverges: considered safe and effective in terms of %EWL by some [21], LPR was not found to offer any major therapeutic benefit by others [14].

In the present study, we retrospectively evaluated weight loss and complication rates after LPR in a consecutive series of bariatric surgery patients undergoing revisional surgery for failure of weight loss.

## Material and Methods

Data from all patients undergoing revisional bariatric surgery were reviewed. Out of 170 revisional bariatric procedures, 32 LPR (27/5, F/M) were performed for failure of weight loss (and dilated gastric pouch) after RYGB and form the study population.

Patient demographics, comorbidities, indication for revision, preoperative weight and BMI, operative time, hospital stay, conversion rate after LPR, overall mean follow-up, body mass index (BMI) loss, percentage excess weight loss (%EWL), indications for surgery, reoperation rate, morbidity, and mortality were reviewed and compared.

### Preoperative Assessment

All patients underwent preoperative routine blood tests, ultrasound examination (US) of the liver and gallbladder, barium upper gastrointestinal (GI) contrast studies and gastroscopy to evaluate gastric pouch for dilatation, stoma size, and the presence of gastro-gastric fistula. Patients were offered surgical correction if the pouch was larger than 30 cc, or the stoma is wider than 1.5 cm, as assessed by radiology and gastroscopy, or the upper part of the pouch was visible during endoscopic retroversion. If gallstones were found, cholecystectomy was performed at the time of revision for both symptomatic and asymptomatic patients. Patients with fatty liver found on US examination were put on high-protein, low-carbohydrate diet 2 to 4 weeks prior to surgery to help reduce liver size. All patients who were positive for *Helicobacter pylori* were treated preoperatively with appropriate antibiotics. Sleep apnea was searched for routinely.

### Surgical Technique

All patients received preoperative low molecular weight heparin in addition to continuous pneumatic compression stocking application during surgery. Prophylactic antibiotics were given preoperatively and continued until patient discharge.

### LPR

Laparoscopic pouch resizing was performed using a 4–5 port technique (5–12 mm, Excell, Ethicon Endosurgery, Cincinnati, OH, USA). Pneumoperitoneum was established with a Veress needle inserted in the left subcostal area. The left liver lobe was retracted by the shaft of a 5-mm grasper, placed via a 5-mm trocar inserted in the subxiphoid position.

**Fig. 1:**
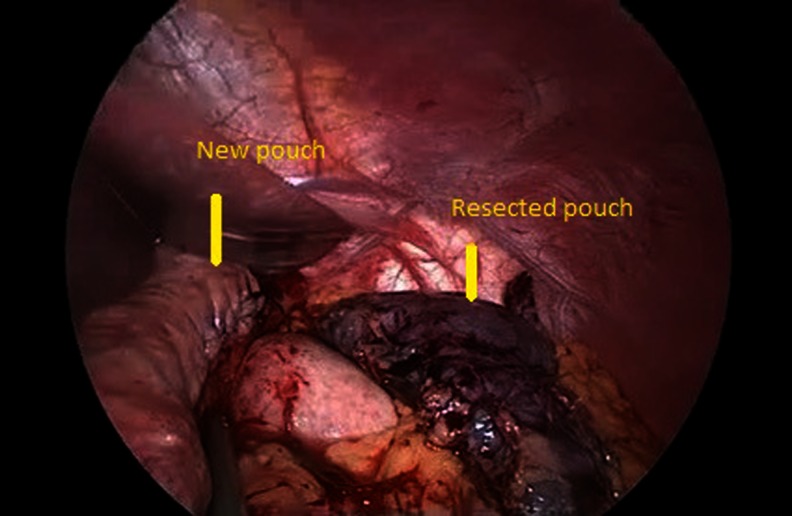
New pouch and resected pouch

**Fig. 2:**
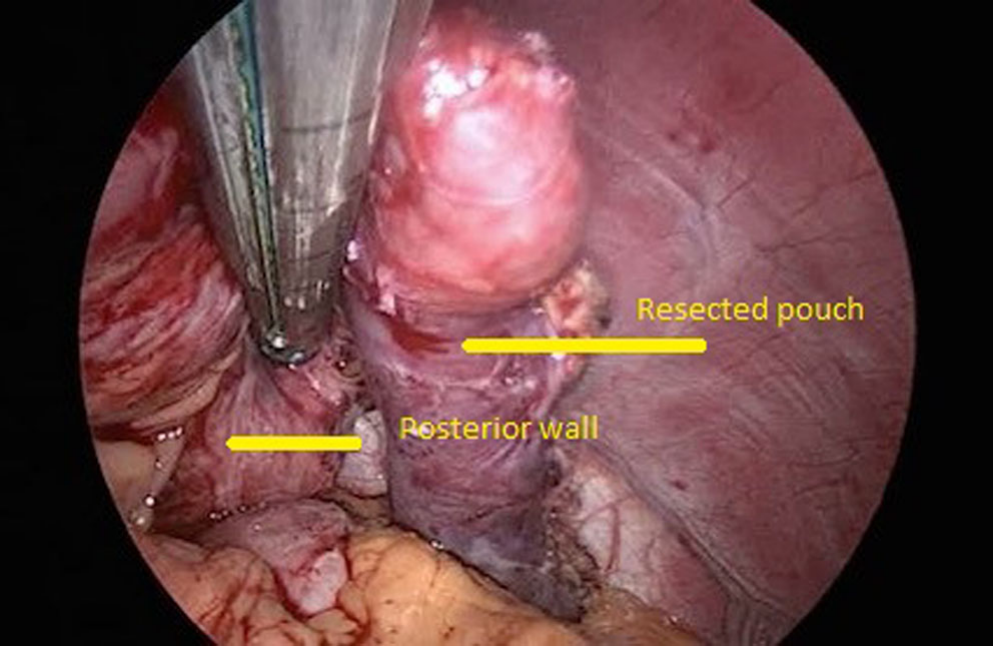
Resected pouch and posterior wall

**Fig. 3:**
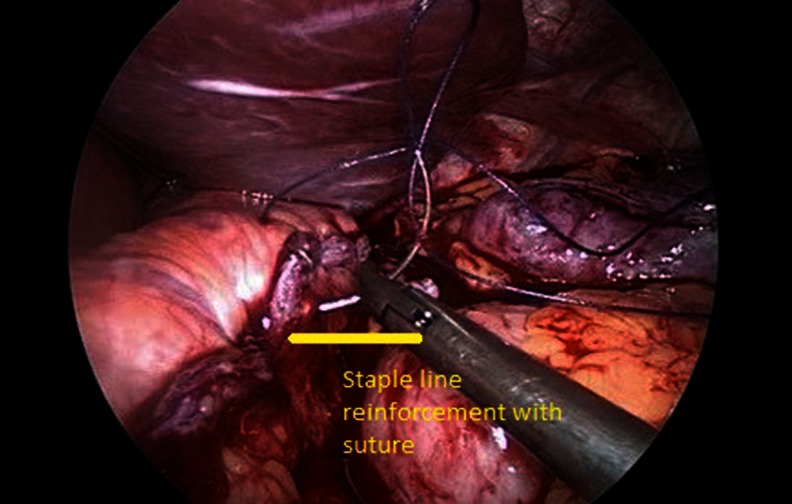
Staple line reinforcement with suture

Adhesions between the gastric pouch, Roux-en-Y limb, and undersurface of the liver were dissected completely. The posterior wall of the pouch and Roux-en-Y limb were dissected free in the direction of the left crus to avoid leaving a large pouch on the posterior wall. A 36 F bougie was then inserted orally by the anesthetist into the jejunum and under laparoscopic guidance. A new 20–25-cc gastric pouch was created by LPR using serial green loads (60 mm, Ethicon Endosurgery, Cincinnati, OH, USA) starting from the dilated Roux loop and across the gastrojejunostomy and gastric pouch up to the gastroesophageal junction. The restapling included 10 cm of the Roux limb, the gastrojejunal anastomosis, and the dilated gastric pouch (Figs. 1, 2 and 3). One patient with a large pouch leading to regain of weight also had a marginal ulcer. We also included one patient with gastro-gastric fistula because this patient also had weight regain and a large pouch. The gastrojejunostomy anastomosis was taken down for the patient with the marginal ulcer, and the ulcer was excised with LPR, and the anastomosis was refashioned. Furthermore, the patient with gastro-gastric fistula underwent LPR and excision of the fistula complex. The staple line was then oversewn with continuous absorbable sutures in all patients. Methylene blue was injected into the nasogastric tube to check for intraoperative leak in all patients. A closed suction drain was inserted routinely.

Patients were discharged on the second postoperative day if there was no evidence of sepsis and if they tolerated oral intake.

Patients were requested to take proton pump inhibitor treatment postoperatively for 3 months. One week after surgery, all patients were also given multivitamins, calcium, iron, and vitamin B12.

### Study Design

Data were collected and analyzed using SPSS 22.0 for windows (Statistical Package for Social Science, Chicago, IL, USA).

## Results

The median operative time and hospital stay were 100 min (range 50–210 min) and 2 days (range 2–5 days), respectively. None of our patients were converted to open.

The overall complication and reoperation rates were 15.6 and 3.1 %, respectively. One patient sustained a postoperative superficial surgical site hematoma at the stapler port site, requiring operative evacuation and blood transfusion under general anesthesia. One patient developed postoperative chest infection and was treated with antibiotics. One patient developed an incisional hernia which was repaired at a later stage. Marginal ulcer was seen in one patient postoperatively who responded to conservative medical treatment.

One patient required several reoperations. She presented 1 week postoperatively with intestinal obstruction and port site hernia. CT scan confirmed the clinical findings and revealed left subphrenic collection; however, there was no evidence of leak even after oral contrast study. She underwent laparotomy and was found to have staple line disruption at the distal stomach in addition to port site hernia that caused intestinal obstruction. This patient had required partial resection of the distal stomach which was seen to be poorly vascularized at the end of LPR procedure. The left subphrenic collection was drained. The pouch staple line was inspected, and there was no leak. She was discharged from the hospital with wound infection. One month later, she presented with fever and sepsis. CT scan revealed a left subphrenic collection, and oral contrast study did not show evidence of leak. It was drained percutaneously. However, 2 days later, she required surgical exploration for pouch leak, where only two drains were inserted. A few days later, a stent was inserted by the gastroenterologist and was kept for 8 weeks. Nasojejunal tube feeding was given until the stent was removed. She developed minor gastrocutaneous fistula that healed after conservative treatment. There was no mortality in our study group. Compliance with postoperative vitamin intake was seen in 57.1 % after LPR (Table 1).

**Table 1:** Conversion, complications, and reoperation

Clinical characteristics	LPR	
	*n*	%
Operative time (min) (median, range)	100	50–210
Hospital stay (days) (median, range)	2	2–5
Conversion	0	0.0
Complications rate	5	15.6
Reoperation rate	1	3.1
Complications		
Wound hematoma	1	3.1
Leak	1	3.1
Incisional hernia	1	3.1
Chest infection	1	3.1
Marginal ulcer (post-LPR)	1	3.3
Compliance with vitamin intake	16/28	57.1

Ten out of 32 patients had obesity-related comorbidities prior to RYGB, including type II diabetes mellitus (TIIDM), hypertension, and obstructive sleep apnea (OSA). All patients with hypertension (2/2) and OSA (1/1) resolved after the primary RYGB. TIIDM resolved in only three (out of four) patients; one patient was still on treatment at the time of LPR. He was lost to follow-up after LPR. Of three patients with bronchial asthma that did not resolve after RYGB, one (1/3) resolved after LPR.

The mean age, preoperative weight, and BMI prior to LPR was 38.3 ± 9.3 years, 101.7 ± 22.8 kg, 38.8 ± 6.4 kg/m^2^, respectively (Table 2). The initial weight and BMI prior to primary RYGB was 133.3 ± 24.6 and 50.7 ± 7.4 kg/m^2^, respectively. The total weight loss and BMI reduction after the primary gastric bypass were 31.6 kg and 12.4 kg/m^2^, respectively, and the median %EWL was 42.6 % (range 1.1–75.5).

**Table 2:** Demographic data

Clinical characteristics	LPR	
	*n*	%
Age (years) (mean ± SD)	38.3	±9.3
Gender		
Male	5	15.6
Female	27	84.2
Preoperative weight (kg) (mean ± SD)	101.7	±22.8
Preoperative BMI (kg/m^2^) (mean ± SD)	38.8	±6.4
Comorbidities		
Diabetes mellitus (pre-RYGB)	4	12.5
Hypertension (pre-RYGB)	2	6.3
Obstructed sleep apnea (pre-RYGB)	1	3.1
Bronchial asthma (pre-RYGB)	3	9.4
Indications		
Inadequate weight loss/weight regain	30	93.8
Marginal ulcer (pre-LPR)	1	3.1
Gastro-gastric fistula	1	3.1

After a mean follow-up of 14.1 ± 6.2 months after LPR, the mean postoperative weight and BMI were 87.8 ± 21.8 and 32.8 ± 7.3 kg/m^2^, respectively, and the median %EWL was 29.1 % (range 0–99 %) (Table 3). Thus, the total weight loss and BMI reduction after the LPR were 13.9 kg and 5.5 kg/m^2^, respectively, and the total median %EWL from the primary RYGB until after LPR was 71.7 %.

**Table 3:** Follow-up

Overall follow-up (months) (mean ± SD)	14.1 ± 6.2
Overall follow-up weight (kg) (mean ± SD)	87.8 ± 21.8
Overall follow-up BMI (kg/m^2^) (mean ± SD)	32.8 ± 7.3
Overall follow-up %EWL (median, range)	29.1 % (0–99 %)

## Discussion

While the literature differs as to the benefits of pouch resizing [11, 21–27], we have shown that LPR leads to a median %EWL of 29.1 % and that the total median %EWL from the primary RYGB until after LPR was 71.7 %.

Failure of weight loss or weight regain after bariatric surgery is multifactorial. Nutritional and psychological factors should be excluded prior to anatomic evaluation. Pouch size, gastrojejunal anastomosis diameter, and length of biliopancreatic and alimentary limbs are the most important anatomic causes. Because the malabsorptive component of RYGB undergoes adaptive mechanisms over time, it has been shown that there was no significant difference between short and long limb RYGB after long-term follow-up [7]. Other authors have shown that elongating the Roux limb corrected failed weight loss in super obese patients but required nutritional support to prevent protein-calorie malnutrition, iron and vitamin deficiency [12, 13]. Furthermore, Näslund showed almost 30 years ago that patients after gastroplasty with smaller stoma lost more weight than those with larger stoma while after gastric bypass, no such correlation was found [22].

Different techniques have been suggested to assess the size of the gastric pouch, but no ideal method has yet been defined [21, 23–25]. In our series, we preferred to use endoscopic and contrast study assessment (>30 cc, or stoma wider than 1.5 cm, or the upper part of the pouch visible during retroversion). We found this method of assessment easy and practical to follow.

Due to the major complications associated with reoperative surgery, a transoral method to treat failure of weight loss or weight regain could potentially eliminate the risks of anastomotic leak and bleeding. Mikami et al. performed Stomaphyx™ on 39 patients and reported 17.0 % (14/39) and 19.5 % (6/39) EBWL at 6 months and 1 year, respectively, without morbidity. However, many patients were lost to follow-up at 6 months and 1 year. Resolution of diarrhea was seen in three patients, and improvement of gastroesophageal reflux was seen in eight patients (19). Ong’uti et al. performed Stomaphyx™ on 27 patients. Only 18/27 patients had >6-month follow-up. The median %EWL was <47 %, and most of their patients reached the maximum weight loss at 6 months, reaching a plateau or beginning to regain some weight after 6 months. Twelve of 14 (86 %) of their patients regained weight by the end of the first year, and they concluded that initial weight loss could have been due to diet modification and close monitoring [20]. A recent review has shown that while these procedures could be of interest in certain cases, their efficacy is limited, and most of the devices are short lived and no longer available [26].

Our technique of LPR is similar to what has been described earlier in the literature. We resize the alimentary limb, gastrojejunostomy, and gastric pouch over a calibration tube without having to redo the anastomosis to avoid the risk of anastomotic leak [14]. Iannelli et al. did not refashion the gastrojejunal anastomosis because they performed a tightly calibrated anastomosis (10 mm) at primary surgery, and during endoscopy at the time of revision, the stoma was <15 mm [21]. They showed that pouch resizing after RYGB was feasible laparoscopically in 90 % of patients and the EWL was 75, 72.2, and 68.6 % at 6-, 12-, and 18-month follow-up, respectively. They also showed that patients with primary dilated pouch had significantly better results than patients with secondary dilated pouch. However, their complication rate was 30 % (21). Parikh et al. reported poor results at 1-year follow-up [14]. The BMI decrease was 2.7 kg/m^2^, and EWL was 12.8 % and even after separately evaluating those patients who underwent Roux limb lengthening (5/14 patients). Furthermore, the bougie size used ranged between 32Fr-60 Fr, and this may explain the low decrease in BMI and EWL. Müller et al. demonstrated the feasibility of laparoscopic pouch resizing and redo pouch-jejunal anastomosis with low morbidity in five patients. The median BMI decreased from 32.0 to 28.0 kg/m^2^ at a median follow-up of 12 months, and diabetes improved in four patients [25]. Hamdi et al. have shown statistically significant weight loss at 3, 6, 9, and 12 months after revisional surgery for gastric pouch and gastrojejunal anastomosis in patients with weight regain after gastric bypass. However, there was no statistically significant weight loss at 24 months in spite of BMI reduction from 54.6 to 44.2 kg/m^2^ [27]. In our study, BMI reduction after LPR dropped from 38.3 to 32.8 kg/m^2^ over a mean follow-up of 14.1 months. Our median %EWL was 29.1 %. We believe that it is crucial to dissect the posterior wall of the gastric pouch from adhesions, since it tends to dilate over time and to use lower bougie size (36 Fr) (reviewer no. 1). Since most of our patients were carbohydrate eaters, we did not lengthen the Roux limb because of concerns about protein malnutrition. Furthermore, compliance with vitamin intake was 57.1 %.

Our morbidity rate after LPR was consistent with other published series [27]. We had no leaks attributable to LPR although one patient had a complicated course secondary to port site hernia and intestinal obstruction that led to staple line dehiscence at the remnant distal stomach after LPR.

However, our results must be interpreted with caution. Our series is relatively small, retrospective, monocentric, with short follow-up.

In conclusion, the exact reasons for weight loss failure after Roux-en-Y gastric bypass remain incompletely elucidated and are probably multifactorial. There is a need to determine the best way to evaluate pouch size as well as the long-term outcome of LPR, as compared to other techniques after Roux-en-Y failure such as Roux limb lengthening, placing an adjustable gastric band on the pouch, or converting the patient to a BPD-DS [12, 13. 14, 15]. Proper evaluation of all therapeutic modalities to correct Roux-en-Y failure (including LPR) remain. Proper preoperative vitamin and mineral intake evaluation is essential if limb lengthening is considered. Furthermore, patients should be informed well about possible protein-calorie malnutrition risks following such procedure. Last, precisions as to Roux limb length at initial surgery might also be important to consider.
